# Efficacy and safety of ozone injection into the intervertebral foramen for treating patients with chronic, intractable postherpetic neuralgia: a one-year follow-up study

**DOI:** 10.3389/fneur.2025.1602689

**Published:** 2025-05-19

**Authors:** Jiang-Lin Wang, Hai-Li Li, Xiang-Bo Liu, Jia-Gui Zhao, Dong Huang, Cheng Wu, Jia-Shuang Wang, Jun Chen

**Affiliations:** ^1^Department of Pain Management, The Affiliated Hospital of Southwest Medical University, Luzhou, Sichuan, China; ^2^Institute for Biomedical Sciences of Pain, Tangdu Hospital, The Fourth Military Medical University, Xi’an, Shaanxi, China; ^3^Department of Anesthesiology, Tianfu Hospital Affiliated to Southwest Medical University, Meishan, Sichuan, China; ^4^Department of Pain Management, The Affiliated Hospital of Chengdu University of Traditional Chinese Medicine, Chengdu, Sichuan, China; ^5^Department of Pain Medicine, The First Affiliated Hospital of Anhui Medical University, Hefei, China; ^6^Department of Pain Management, The Third Xiangya Hospital of Central South University, Changsha, Hunan, China; ^7^Department of Anesthesiology, Hejiang People’s Hospital, Luzhou, Sichuan, China; ^8^Department of Pain Medicine, Guangzhou Red Cross Hospital, Jinan University, Guangzhou, Guangdong, China; ^9^Department of Pain Medicine, Jinshazhou Hospital of Guangzhou Traditional Chinese Medicine, Guangzhou, Guangdong, China; ^10^Sanhang Institute for Brain Science and Technology, School of Medical Research, Northwestern Polytechnical University, Xi’an, Shaanxi, China

**Keywords:** herpes zoster, postherpetic neuralgia, spontaneous pain, hyperalgesia, intervertebral foramen injection of ozone (IVFO), infrared thermography, gabapentin

## Abstract

**Introduction:**

Chronic intractable postherpetic neuralgia (PHN) is a significant sequel of herpes zoster and significantly impacts patients’ quality of life. Although some pharmacotherapies, interventional approaches, and neural modulation have been recommended as clinical options, their efficacy is limited. Here, we reported the efficacy and safety of a standardized therapeutic approach with CT-guided intervertebral foramen injection of ozone (IVFO) in patients with chronic intractable thoracic and lumbar (PHN) (*n* = 56) who had been tolerant or insensitive to first-line drugs, such as gabapentin (GBP) or pregabalin.

**Methods:**

Visual analogue scale (VAS), quantitative sensory testing (von Frey filaments only), and infrared thermography were used to identify and quantify the pain intensity, area of mechanical hyperalgesia, and skin temperature in the included patients with PHN before and after IVFO treatment. Moreover, the dosage of and the time to discontinue GBP and complications were also documented after discharge from hospitals.

**Results:**

In this 1 year follow-up study, the primary endpoint outcomes measured by VAS showed that IVFO treatment resulted in significant relief of spontaneous pain by 59.19% [2.67 ± 0.66] for immediate, 68.18% [2.08 ± 0.89] for half year and 70.79% [1.91 ± 1.19] for 1 year after discharge vs. admission, dramatic decrease in spatial area of mechanical hyperalgesia by 52.35% [3.11 ± 0.70] for immediate and 87.41% [0.82 ± 0.50] for half year after discharge vs. admission and skin temperatures by 63.01% [0.85 ± 0.35] for immediate after discharge vs. admission. Moreover, half of the patients stopped taking GBP 3 months after discharge. No serious complications were reported during the one-year follow-up after IVFO treatment.

**Conclusion:**

These results suggest that CT-guided IVFO treatment is a safe and effective interventional approach for the relief of chronic, drug-resistant, thoracic and lumbar PHN.

## 1 Introduction

Herpes zoster (HZ) is an inflammatory skin response caused by the restimulation of varicella-zoster virus (VZV) in the corresponding innervation area of the spinal nerves (1). Several studies have shown that the incidence of HZ is directly correlated with age; the incidence of HZ will quickly rise after 50 the age (2). A systematic review suggests that the incidence rate of HZ ranged between 3 and 5 /1,000 people per year, among which 5 to 30% of patients have developed postherpetic neuralgia (PHN) (3–5). PHN is the most common and the most serious sequelae of HZ. Most patients complain of an ongoing or intermittent spontaneous pain of, for example, burning, pricking, squeezing quality, which may be accompanied by evoked pain, particularly to light touch and cold; what’s worse, some people can also be accompanied by the corresponding motor nerve dysfunction. Long-term severe pain greatly affects the patients’ quality of life, and brings a heavy burden to the family and society (6).

Currently, in addition to oral drug analgesia, the treatment of PHN usually involves pulsed radio frequency, intrathecal infusion system implantation, peripheral nerve or spinal cord electrical stimulation, and other nerve regulation techniques, as well as spinal dorsal root gangliectomy (7–10). Due to the pathogenesis not having been fully elucidated, although there are many treatment methods, there is no effective cure, and all of them have some side effects. Nowadays, medical ozone (O_3_) has been widely used in pain treatment because of its strong antioxidant and analgesic action (11, 12). Certain concentration (≤30 ug/ml) of O_3_ can effectively eliminate the pain-causing factors and adhesions around the dorsal root ganglion (DRG), spinal nerve roots and sympathetic ganglion, improve local hypoxia state, and promote the damage of nerve repair process, at the same time, vascular endothelial cells are stimulated to release NO and platelet-derived growth factor (PDGF), which can cause vascular dilation and improve local microcirculation, thus promoting inflammation absorption (12, 13). As a consequence, we aim to report to a group of patients with PHN receiving standardized low-concentration O_3_ therapy for the treatment of refractory PHN. We have obtained preliminary data on the feasibility, safety, and clinical efficacy of O_3_ therapy for the treatment of PHN, which has laid a solid foundation for further studies about the treatment of PHN.

## 2 Methods

### 2.1 Participants

This is a prospective, multi-center clinical trial. The protocol followed the SPIRIT clinical trial guidelines (14), and all procedures for human patients involved in the study were in accordance with the ethical standards of the Helsinki Declaration and its later amendments. The protocol of this study has been approved by the Ethics Committee of Guangzhou Red Cross Hospital affiliated of Jinan University (with a registration number 2014-078-02) and registered in the Chinese Clinical Trial Registry. All participants have signed the informed consent.

**Figure 1:**
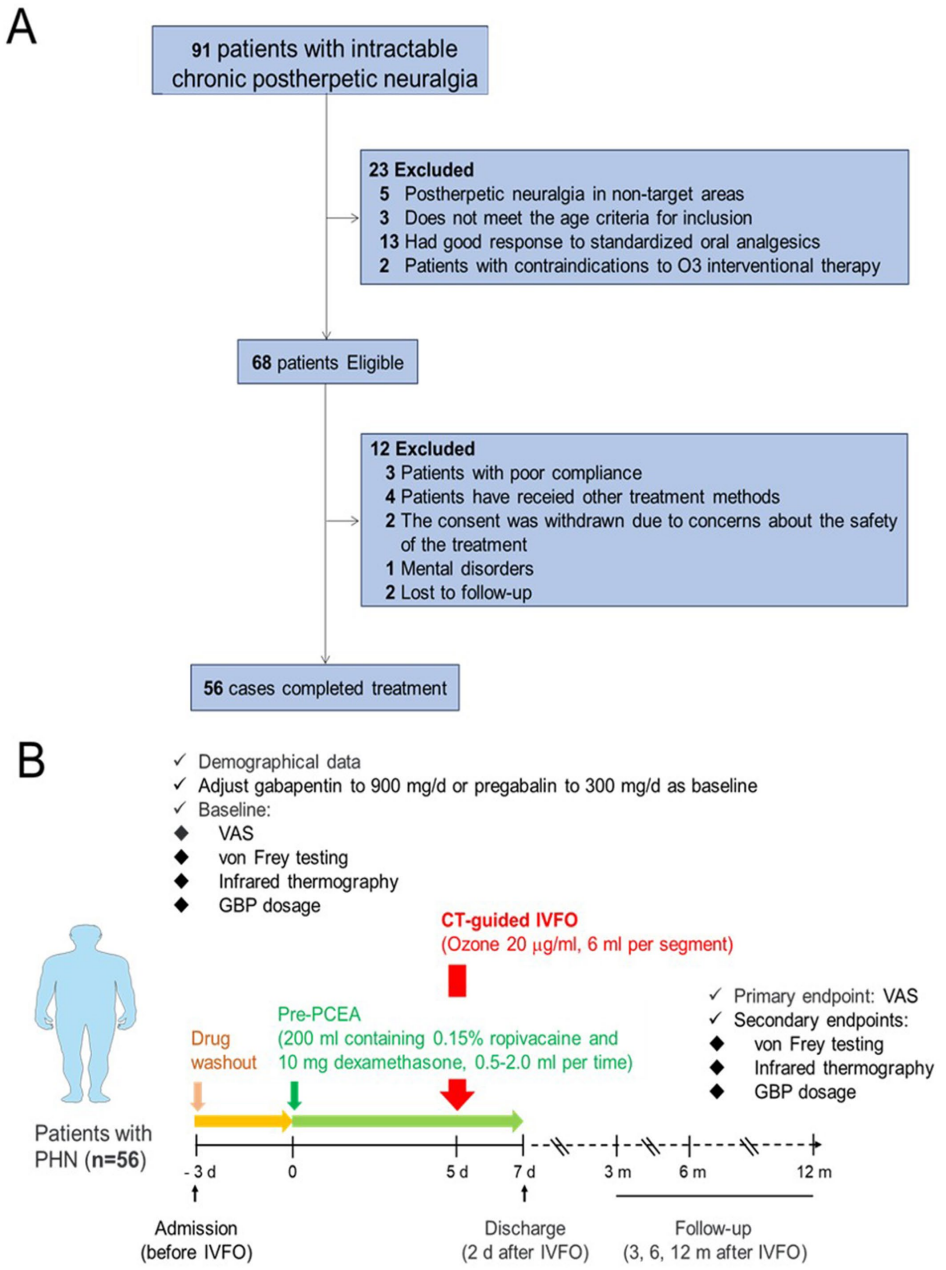
A flowchart of patient inclusion and exclusion is shown in panel **(A)**, and the time flow for IVFO treatment is shown in panel **(B)**.

This study included patients with thoracic and lumbar postherpetic neuralgia who were admitted to the hospital from 2012 to 2019. Inclusion criteria: (1) Patients have a history of acute herpes zoster, persistent or recurrent pain for more than 3 months after healing of skin lesions, and obvious postherpetic pigmentation in the area of thoracic and lumbar innervation (T1 ~ L5). (2) The pigmentation area after HZ is accompanied by severe spontaneous or paroxysmal pain and skin irritation. The nature of the pain, such as knife cutting, electric shock, burning, or acupuncture, is accompanied by pruritus, ant travel, or a tight feeling. (3) All patients had poor response to standardized oral analgesics (including gabapentin or pregabalin, non-steroidal analgesics, or antidepressants, etc.) or could not tolerate drug side effects. (4) Elderly patients who meet the inclusion criteria and are between 50 and 85 years old. Exclusion criteria: (1) Do not meet PHN diagnostic criteria. (2) Combined with other underlying diseases causing neuralgia, the pain could not be clearly identified as the cause of PHN alone. (3) Patients with contraindications to O_3_ interventional therapy, such as site infection, inflammation, or severe systemic infection. (4) Complicated with serious cardiovascular, cerebrovascular, liver, kidney, hematopoietic system, and other primary diseases and malignant tumors. (5) Patients with critical or mental disorders make it difficult to evaluate the effectiveness and safety of treatment. Expulsion case criteria: (1) Enrolled but did not complete the treatment regimen. (2) Patients who have been enrolled and completed the treatment regimen, but have received other treatment methods. (3) PHN patients who refused regular detailed post-treatment evaluation and follow-up (Figure 1A).

### 2.2 Description of interventions

Patients with intractable pain have been on multiple medications before, all patients need to adjust the drug category and dosage after enrolment. We defined this period as the drug baseline period (Oral medications: mecobalamin 1.5 mg/day, gabapentin 300 ~ 900 mg/day, or pregabalin 150 ~ 300 mg/day). Patient-controlled epidural analgesia (PCEA) was administered (0.15% ropivacaine and 10 mg dexamethasone total 200 mL, maintenance dose was 0.5 ~ 2 mL/h, single additional dose was 0.5 ~ 2.0 mL, 20 ~ 40 mL/day), PCEA was administered continuously for 1 week, and ozone interventional therapy was performed under the guidance of CT on the 5th day (Figure 1B).

#### 2.2.1 PCEA therapy

**Figure 2:**
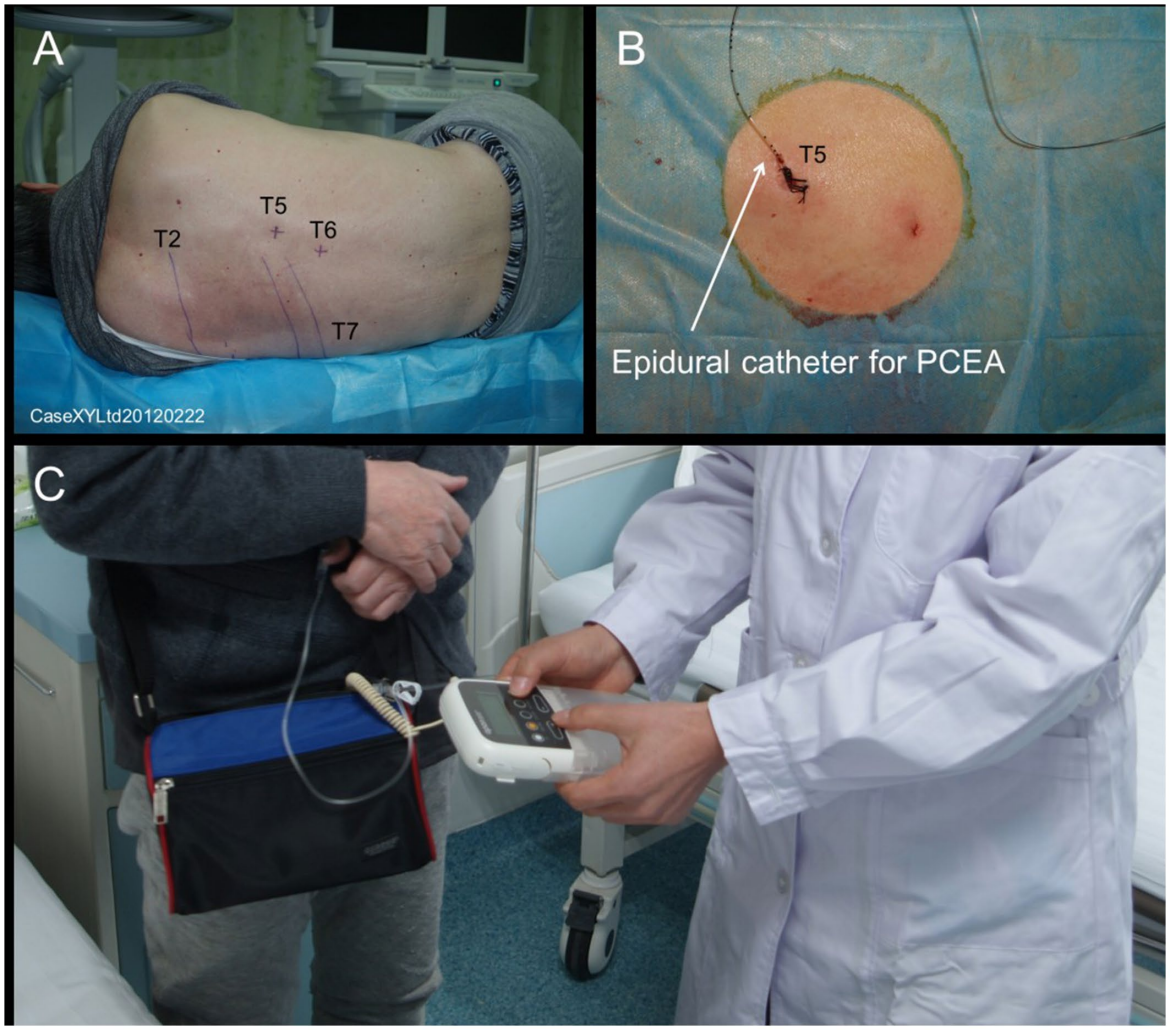
The patient’s position during PCEA therapy is shown in panel **(A)**; Panel **(B)** shows the epidural catheter for PCEA. Panel **(C)** shows the regulatory process during PCEA therapy.

The puncture location was the spinous process space corresponding to the spinal nerve segment of the primary herpes injury. The puncture process is strictly aseptic, and damage to blood vessels and nerves should be avoided. After a successful puncture, the epidural catheter was inserted through a subcutaneous tunnel of about 5 cm and then fixed to the skin. At the same time, appropriate prophylactic antibiotics can be used throughout the treatment process of PCEA according to the patient’s condition to prevent secondary infection. The IVFO approach was performed after 5 days of continuous PCEA administration (Figure 2).

#### 2.2.2 Intervertebral foramen injection of ozone (IVFO) approach

**Figure 3:**
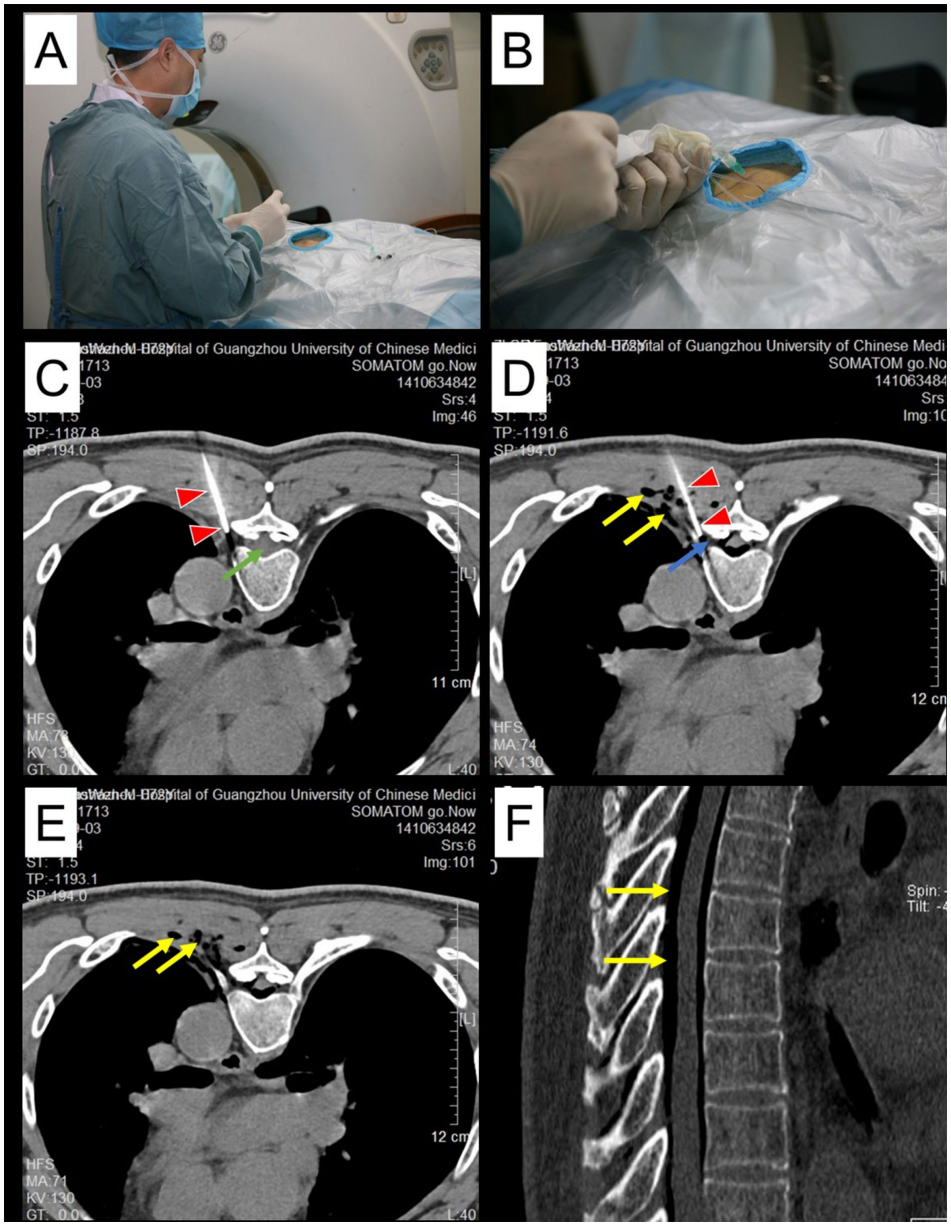
A case showing transverse and sagittal images of a CT scan taken immediately after an IVFO ipsilaterally to the infected segments of PHN. The doctor performed the puncture operation, as shown in panels **(A, B)**. Double arrowheads (red color) indicate the tip site and track of the puncture needle **(C, D)**. The double arrows (yellow) represent ozone (D-F). Ozone is distributed in the gas distribution around the dorsal root ganglion (**D**, blue arrow) and the epidural space **(F)**. The green arrow shows the spinal cord.

Patients’ basic vital signs were monitored dynamically. Patients with poor cardiopulmonary function inhaled oxygen continuously at low flow to ensure their’ vital signs were stable. The IVFO approach was carried out under CT guidance in order to improve the success rate of the puncture. The operation was carried out after the puncture needle was connected with the extension tube and syringe to prevent accidental injury to the pleura. After no blood or fluid was extracted, O_3_ was injected at a concentration of 30 μg/mL, and the dose was 8 ~ 12 mL/foramen. Two discontinuous foramens were punctured at one interval each time (such as T3 and T5; T4 and T6) (Figure 3).

### 2.3 Evaluation of therapeutic effect

#### 2.3.1 Assessment of pain and nerve damage

**Figure 4:**
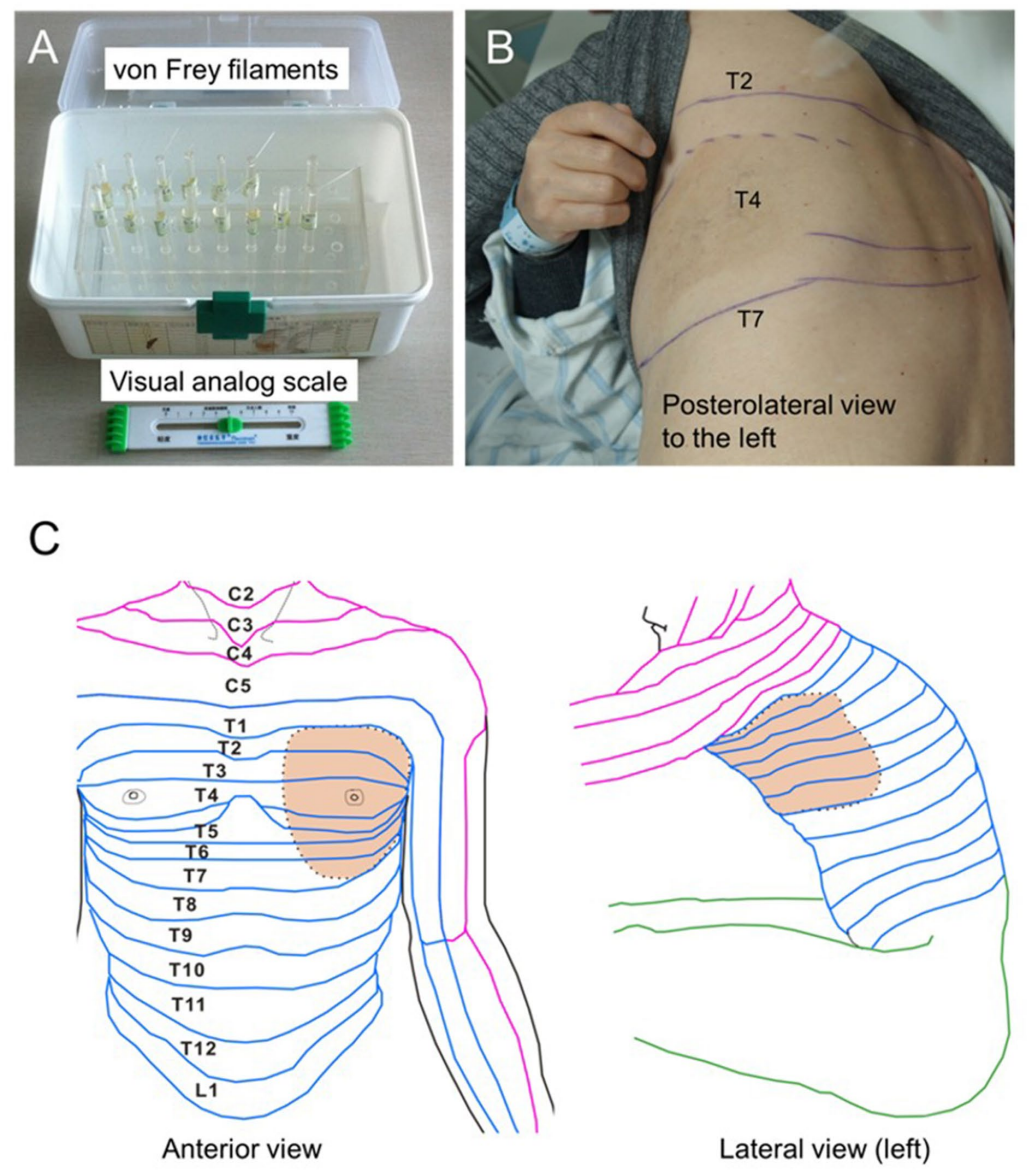
The pain degree of the patient was quantified using Von Frey filament and visual analog scale **(A)**. Panels **(B,C)** showed the lesion area of herpes zoster, respectively.

The primary and secondary lesions, as well as the damaged spinal nerve segments, were measured at admission and discharge after treatment using sterile cotton swabs and mechanical Von Frey filament (VFF) (Figures 4A,B). Infrared thermography was used to monitor the changes in skin temperature in the primary and secondary injury areas. This method can qualitatively and quantitatively detect the abnormality of sympathetic nerve function and judge the occurrence of neurogenic inflammation. Patients’ visual analogue scale (VAS) was recorded via a 10-centimeter-long ruler at admission, discharge, half a year, and 1 year after discharge. Patients were instructed to exhibit their pain level via the VAS ruler immediately before the first assessment, where higher scores suggest higher pain levels.

#### 2.3.2 Assessment of clinical efficacy and post-treatment complications

The dosage of anti-neuropathic pain drug (gabapentin) was adjusted to 600 mg/d at discharge, and the time when the patients stopped using gabapentin due to pain relief was recorded. Any adverse events related to the IVFO approach (such as infection, spinal anesthesia, pneumothorax) during treatment were recorded.

### 2.4 Statistical analysis

All data were analyzed using SPSS Version 25.0 (SPSS Inc., Chicago, IL, USA). The measurement data of skew distribution were expressed as median and interquartile range [M(Q)], and the measurement data of normal distribution were expressed as Mean ± SD. The independent sample *t-test* was used to test the difference between participants. A *p*-value less than 0.05 was considered significant.

## 3 Results

### 3.1 Patient demographics

**Figure 5:**
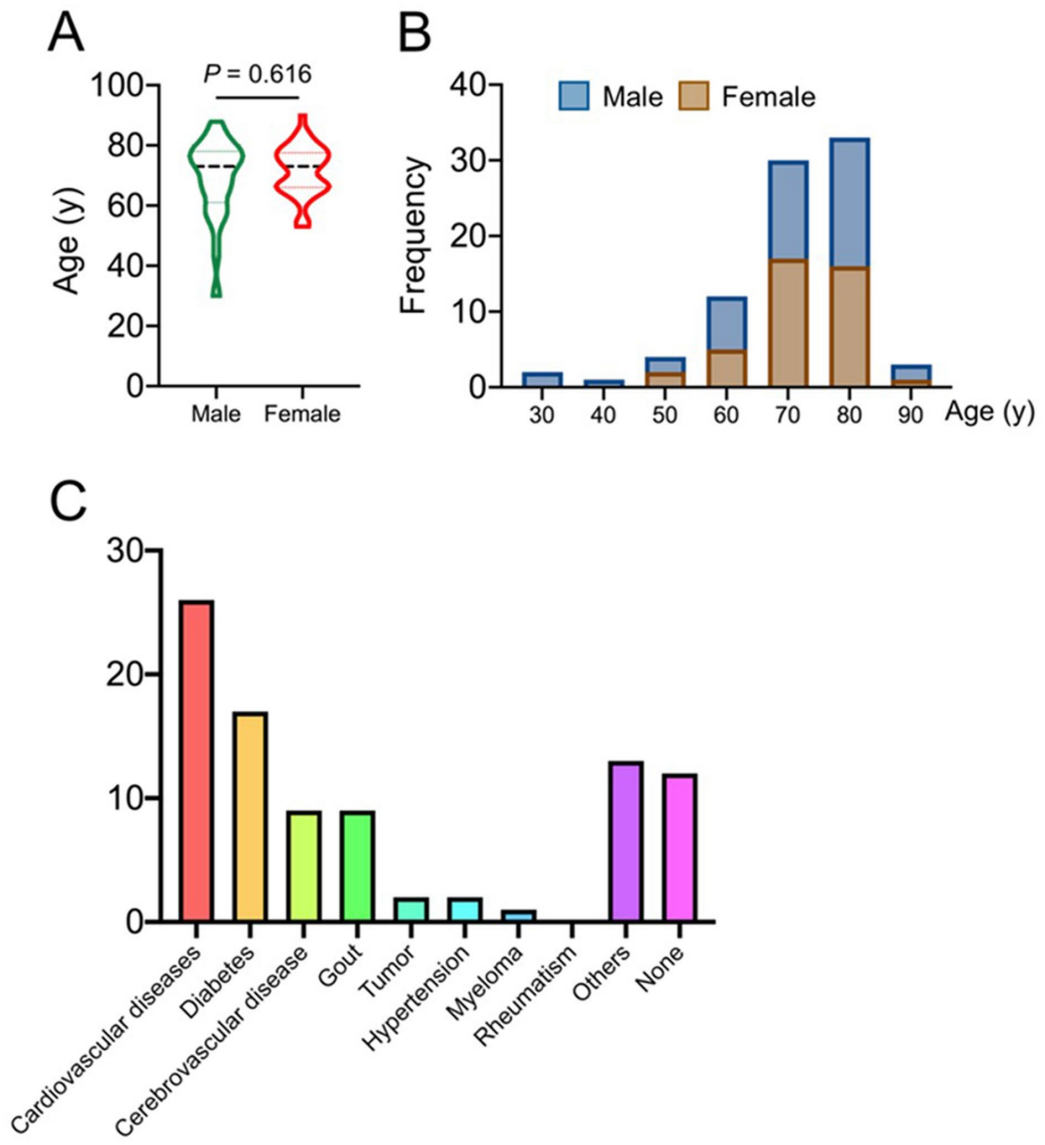
Epidemiological characteristics as shown in panels **(A, B)**, and panel **(C)** shows the comorbidity of postherpetic neuralgia patients.

A total of 56 patients were involved in this study, and their baseline data (such as gender, age, education level, pain duration, etc.) were listed in Table 1. Figures 5A,B show the correlation between age of onset and gender in postherpetic neuralgia. The underlying diseases that coexisted with the participants were listed in Figure 5C.

**Table 1:** Epidemiological characteristics of postherpetic neuralgia.

Condition of the newly diagnosed patients		Total
Age, mean±SD		69.75 ± 11.4
Gender, *N* (%)	Male	21 (37.50)
	Female	35 (62.50)
Registered residence, *N* (%)	Urban	47 (83.93)
	Rural	9 (16.07)
Education *N* (%)	≤6 years (Junior school)	14 (25.00)
	>6 & ≤ 12 years (High school)	23 (41.07)
	>12 years (College or higher level)	19 (33.93)
Marital status, *N* (%)	Married	50 (89.29)
	Divorced	0 (0.00)
	Widowed	6 (10.71)
	Unmarried	0 (0.00)
	Separated	0 (0.00)
Work status, *N* (%)	Employed	8 (14.29)
	Unemployed	48 (85.71)
Allergic history, *N* (%)	No	51 (91.07)
	Yes	5 (8.93)
Family history, *N* (%)	No	56 (100.00)
	Yes	0 (0.00)
History of drinking, smoking and drug, *N* (%)	No	31 (55.36)
	Cigarette	19 (33.93)
	Drink	15 (26.79)
	Drug	0 (0.00)
Toxic exposure, *N* (%)	No	56 (100.00)
	Yes	0 (0.00)
Duration of pain (month), mean ± SD		17.53 ± 28.24
Pain area, mean ± SD		2.02 ± 1.76

### 3.2 The damaged nerve

**Figure 6:**
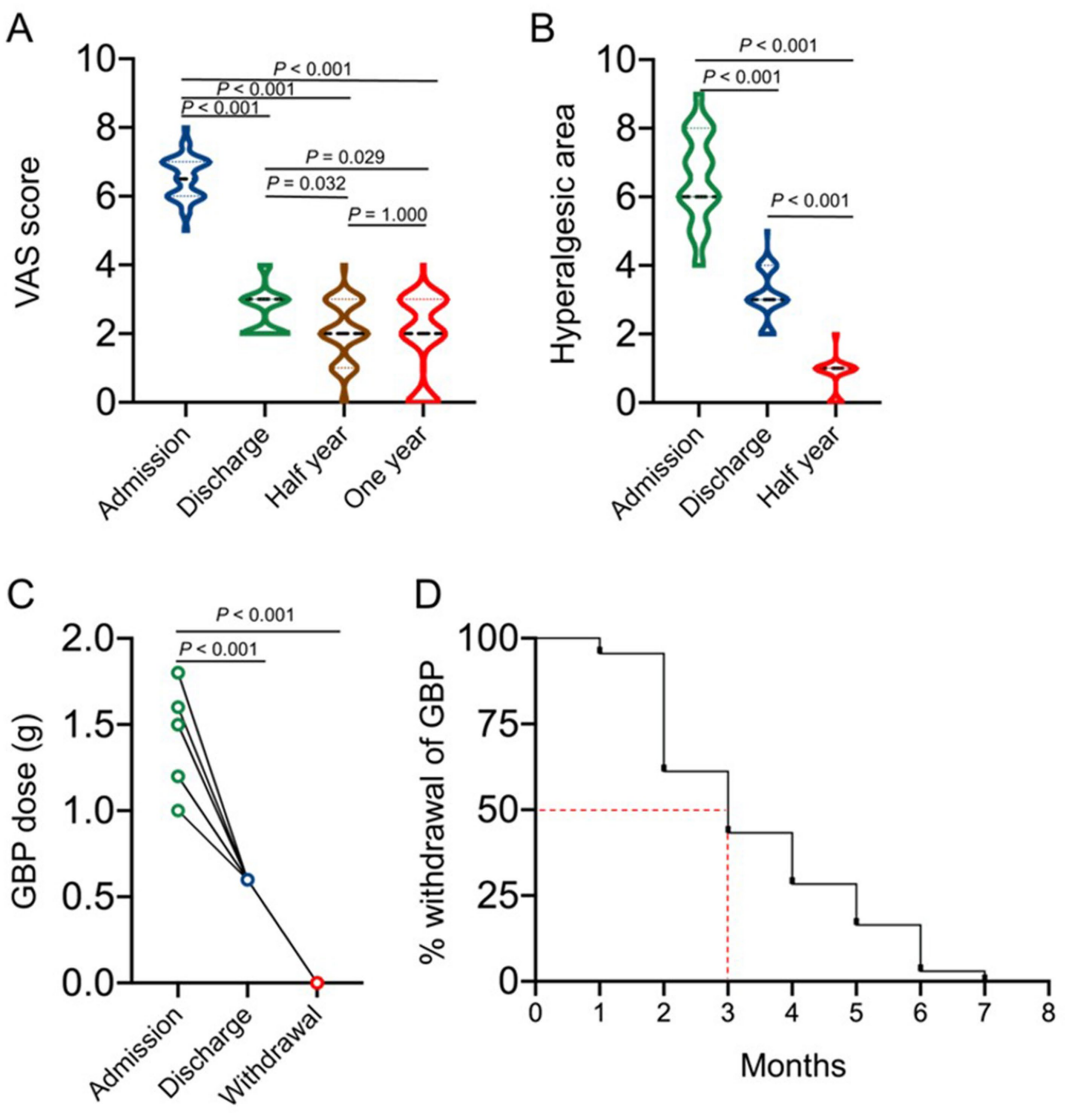
Primary endpoint assessment of the therapeutic effects of the IVFO treatment on the intensity of pain in patients with chronic PHN measured by VAS score, as shown in panel **(A)**. The hyperalgesia area of patients with PHN at admission, discharge, and 3 months after treatment was shown in panel **(B)**. The therapeutic effects of the IVFO treatment on dosages of gabapentin in patients with chronic PHN, as shown in panel **(C)**, and the withdrawal time of gabapentin in patients with chronic PHN is shown in panel **(D)**. Patients consumed less gabapentin (600 mg/day) to maintain the analgesic effect when discharged, while half of the patients would stop medication 3 months after discharge.

**Figure 7:**
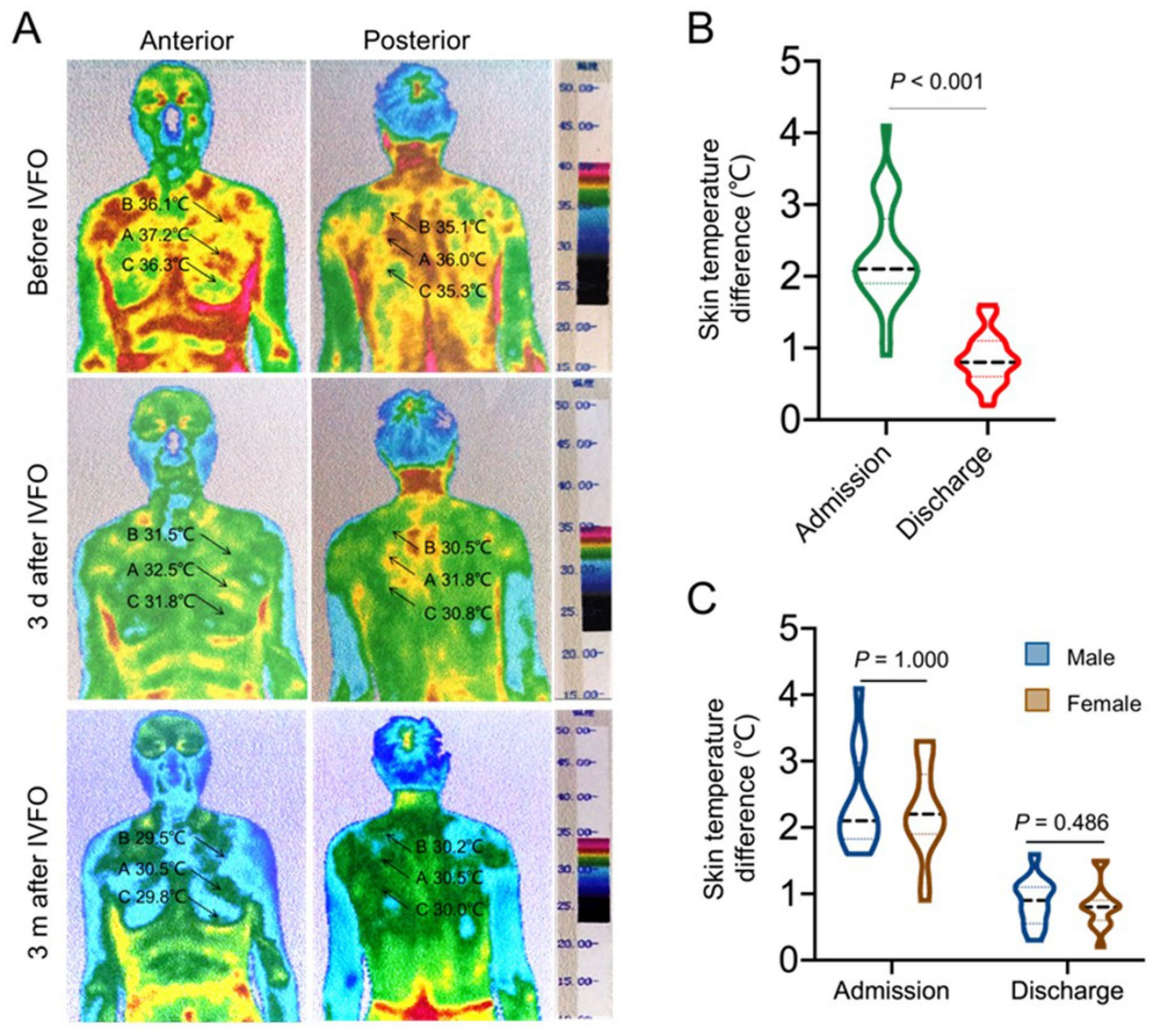
The therapeutic effects of the IVFO treatment on the sympathetic functions in patients with chronic PHN. **(A)** Infrared thermographic images showing improvement of sympathetic functions in patients with chronic PHN before and 3 days and 3 months after IVFO treatment. Panels **(B,C)** showing the quantitative analysis of the therapeutic effects of the IVFO treatment on skin temperature changes.

Areas of primary and secondary nerve injury were significantly reduced and could not be distinguished in patients with PHN at discharge after IVFO treatment (Figure 6B). The infrared temperature difference between the affected and healthy sides of patients with PHN at admission, discharge and 3 months after treatment was shown in Figure 7, which indicated that the damaged area (the sum of the primary and secondary spinal nerve injury segments) at admission was significantly reduced compared with that at discharge and 3 months after treatment. The infrared temperature difference between the affected and healthy sides was significantly reduced at admission, discharge, and 3 months after treatment.

### 3.3 VAS scores and duration of gabapentin

Patients’ VAS scores at admission, discharge, half year as well as 1 year after discharge were shown in Figure 6A, which suggested that the VAS scores of the PHN patients at admission were significantly decreased compared with those at discharge, half year and 1 year after discharge, while the difference between half year and 1 year after discharge was not statistically significant. The dosage of gabapentin was adjusted to 600 mg/ day at discharge, and the results showed that half of the patients stopped medication 3 months after discharge (Figures 6C,D).

### 3.4 Adverse events

No serious complications occurred in all 56 patients, and all participants showed good compliance. Major complications included local pain, numbness in the affected area, dizziness, and nausea.

## 4 Discussion

Most prior studies have primarily investigated the efficacy of pulsed radiofrequency combined with ozone injection for managing intractable postherpetic neuralgia (PHN), with their synergistic therapeutic effects well-documented (15, 16). Our study further clarifies the effectiveness of ozone injection in the treatment of PHN. In our cohort, the epidemiological characteristics of the included patients, such as sex ratio, age, education level, pain duration, and pain area, were comparable. Our findings indicated that, after PHN patients were treated with the IVFO approach, the pain level, pain area, body surface temperature, and time of discontinuation of gabapentin were all chronically improved at each time point after discharge, and no serious complications occurred during the entire treatment period.

According to the European Federation of Neurological Societies (EFNS) and the NEUP Special Interest Group of the International Association for the Study of Pain (IASP), the recommended first-line drugs for the treatment of PHN include calcium channel modulators (pregabalin and gabapentin), tricyclic antidepressants (amitriptyline) and lidocaine patches (17). It is pointed out that the rational use of multimodal analgesia in the treatment of early HZ can effectively relieve PHN in the early stage and prevent PHN (18). Therefore, in our study, we pioneered the comprehensive treatment regime based on the IVFO approach to achieve desirable efficacy in the treatment of PHN. Medical ozone is a mixture of ozone and oxygen. O_3_ is an unstable blue gas at room temperature and has a special pungent smell. Its chemical properties are extremely unstable, and it has a strong oxidation ability. At present, medical ozone is more and more widely used in the field of pain treatment. When using the appropriate concentration of ozone for treatment, it can act as a physiological activator to stimulate many biological effects in the body. Subcutaneous injection of O_3_ in the treatment of PHN can rapidly relieve pain, significantly reduce tissue congestion and edema, reduce local tissue temperature, and increase local tissue oxygen supply. Studies have found that subcutaneous sensory nerve endings (unmyelinated nerve fibers) and skin glial cells (Schwann cells) can form a unique pain sensory organ in the skin, suggesting that changes in skin sensory nerve fiber density or skin inflammatory factors during PHN affect pain perception. In addition to reducing local inflammatory reactions, ozone, due to its dispersity, plays an important role in the release of soft tissue hyperplasia, adhesion, and scars to a certain extent. At the same time, the local nerve endings can also stimulate the body to release endorphins and other substances to block the transmission of harmful signals to the cortex and thalamus, thus producing an analgesic effect (19). Zhou et al. (20) used different concentrations of ozone (20 μg/mL, 40 μg/mL, 60 μg/mL, 80 μg/mL) to intervene astrocytes cultured *in vitro*, and the results showed that high concentration of ozone can damage the activity of glial cells and their mitochondria to a certain extent. The damaging effect of ozone increased gradually with the increase of ozone concentration, indicating that high-concentration ozone has a certain degree of toxic effect on the central nervous system, while there was no obvious damage to astrocytes in the low concentration group (≤ 40 μg/mL group), so in our study we used 30 μg/mL O_3_ concentration to ensure the safety of patients during treatment.

In 2017, Luo et al. conducted a study on the influence of ozone on NP model rats, in which they found that NP rats’ mechanical pain threshold was significantly controlled by administering ozone to L4 ~ 5 intervertebral foramen (21). What’s more, the analgesic duration of ozone treatment was longer than that of other selective molecular target drug inhibitors or antagonists such as NAV1.8 (A-803467), CXCR4 (AMD3100), mTOR (rapamycin), and Histone deacetylase (MGCD0103). Interestingly, in this study, IVF-directed injection of ozone synergistically enhanced the analgesic effect of gabapentin on NP and had no effect on normal rats’ pain threshold. In our study, we also adjusted the dose of anti-neuropathic drugs such as gabapentin for the included patients, and considering the defects of nerve root stimulation and local puncture pain in the IVFO approach, we used PCEA analgesia to overcome them. Under the guidance of CT, precise positioning and O_3_ injection were carried out in the affected side foramen of patients with PHN. It was observed that the local skin temperature, hyperalgesia, VAS scores, and other indicators of patients with PHN were significantly improved before and after treatment, which suggested the feasibility and clinical efficacy of this method.

A meta-analysis suggested that regular medications and early intervention are the best option for treating PHN; the appropriate combination of different interventions could improve pain relief, and clinicians should strive to achieve individualized treatment (22). Some randomized controlled studies have shown that gabapentin or pregabalin, combined with nerve pulsing radiofrequency, nerve block, and percutaneous electrical nerve stimulation, might have a positive effect on patients with PHN (23–25). However, it has some disadvantages, such as repeated treatment and relatively high cost. In general, the IVFO approach, because of its high clinical feasibility, is easy to be accepted by patients and is convenient for clinical application, which can be used as an alternative program for the treatment of intractable PHN.

Nevertheless, our current study design has some limitations that should be addressed in future trials. Firstly, this study was not a double-blind, randomized controlled trial. Secondly, this study only included patients with chest and waist PHN. Thirdly, repeated O_3_ interventions may be required for some patients with a long course of disease. Last but not least, the sample size of this study is small, and future studies need a larger sample size randomized controlled study to further evaluate its benefits and risks.

## 5 Conclusion

CT-guided Intervertebral foramen injection of ozone treatment is a safe and effective treatment for relief of chronic thoracic and lumbar PHN., which can significantly reduce nerve damage, reduce long-term VAS score, and reduce the use of anti-neuropathic pain drugs after discharge.

## Data Availability

The raw data supporting the conclusions of this article will be made available by the authors, without undue reservation.
